# Genome Sequencing and Comparative Genomics of the Hyper-Cellulolytic Fungus *Talaromyces pinophilus* Y117

**DOI:** 10.3390/jof11090614

**Published:** 2025-08-22

**Authors:** Ya Li, Siyuan Yue, Peng Li, Jing Zeng, Jianjun Guo, Dawei Xiong, Shuaiwen Zhang, Tao Deng, Lin Yuan

**Affiliations:** Institute of Microbiology, Jiangxi Academy of Sciences, Nanchang 330095, China; liya0104@126.com (Y.L.); jxskxyysy@126.com (S.Y.); pengli_jx@126.com (P.L.); zengjingwhu@126.com (J.Z.); guojianjunjxas@163.com (J.G.); xiongdawei@163.com (D.X.); zhangsw0714@126.com (S.Z.);

**Keywords:** *Talaromyces pinophilus*, cellulose-degrading fungus, genomic sequencing, comparative genomics

## Abstract

*Talaromyces pinophilus* is a filamentous fungus with notable lignocellulose-degrading capacity based on enzyme activities and protein secretion potential, making it a compelling candidate for industrial biotechnology applications. In this study, we present the genomic characterization of the highly cellulolytic strain Y117, a domesticated variant of *T. pinophilus*, based on whole-genome sequencing and comparative genomic analysis with eleven related strains. Comprehensive analysis of CAZymes, transcription factors, and secondary metabolite diversity in *T. pinophilus* strains revealed that the exceptional lignocellulose degradation capacity of Y117 is driven by its unique genomic architecture. Key genomic features that distinguish Y117 include (1) significant expansion of glycoside hydrolase (GH) and carbohydrate-binding module (CBM) families, (2) loss of fungal-RiPP-like clusters, and (3) absence of the developmental regulator BrlA. These genomic adaptations could indicate a metabolic trade-off favoring hydrolytic enzyme production over secondary metabolism and sporulation. Our findings provide fundamental insights into fungal lignocellulose degradation mechanisms while establishing Y117 as a promising chassis for metabolic engineering applications in industrial enzyme production and heterologous protein expression.

## 1. Introduction

Cellulosic biomass is an abundant, renewable, and underutilized global carbon source [1,2]. It contains a large quantity of sugars in the form of cellulose and hemicellulose, thus holding significant potential for sustainable bioenergy production. Efficient conversion of cellulose and hemicellulose into sugar is the key to fully unlock its energy potential. It has been reported that some filamentous fungi are wood-degrading organisms through secreting large amounts of extracellular cellulases and hemicellulases that can hydrolyze lignocellulosic biomass to fermentable sugars [3]; notably, *Trichoderma reesei* is the leading cellulase producer for biorefinery applications [4]. However, some challenges, such as improving the efficiency of biomass pre-treatment, enzymatic hydrolysis, and conversion technologies, need to be addressed to optimize the utilization of lignocellulosic biomass [5,6]. Ongoing efforts are being made to turn cellulosic biomass into a cornerstone of the renewable energy sector [7,8].

*Talaromyces pinophilus* (*T. pinophilus*), formerly designated as *Penicillium pinophilum*, is a filamentous fungus belonging to the Ascomycota phylum, widely recognized for its ability to degrade lignocellulosic biomass and produce valuable enzymes [9,10]. The fungus thrives in diverse environments and exhibits robust growth under varying conditions, making it a versatile candidate for industrial use. *T. pinophilus* Y-94 was isolated in 1982 from soil in the Tohoku region, Japan, which can secrete large quantities of cellulolytic and hemicellulolytic enzymes [11]. In the pursuit of more economical cellulolytic enzyme production, successive rounds of random mutagenesis were applied to wild-type strain Y-94 to screen for hyper-producing mutants. Among all mutants, CF-2612 emerged as the most efficient cellulase producer, exhibiting significantly superior β-glucosidase secretion capacity that surpassed even that of *Trichoderma reesei* [12,13]. Following these discoveries, researchers have focused on identifying the underlying regulation mechanisms of cellulase expression to further improve their productivity [14,15]. Strategic genetic modifications, including the deployment of enhanced promoter systems and productivity-related transcription factors, have been applied to maximize enzyme yields and functional efficiency for industrial applications [16]. Additionally, *T. pinophilus* exhibits remarkable potential as a cellular factory for the production of various bioproducts, including thermophilic proteins, owing to its robust protein secretion capacity [17,18].

Despite advances in *T. pinophilus* genetic modification, limitations such as low enzymatic efficiency and high production costs remain, hindering its industrial application in the degradation of lignocellulosic biomass [19]. To address these challenges, in-depth genetic investigations are essential to elucidate the comprehensive mechanisms of cellulose degradation in *T. pinophilus*, thereby facilitating its industrial utilization [20]. Although the complete genome of *T. pinophilus* strain 1-95 has been sequenced and its degradation mechanisms have been characterized, this strain’s limited cellulase production makes it unsuitable as a model organism [21]. Moreover, the existing research lacks systematic comparative genomic analyses among different *T. pinophilus* strains, which could provide critical insights into strain-specific degradation capabilities and help identify superior enzyme producers for industrial applications.

**Figure 1:**
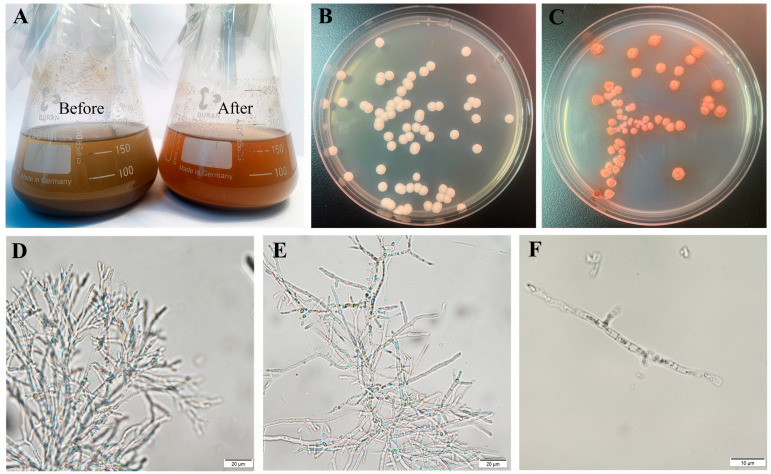
Phenotypic and functional characterization of *T. pinophilus* Y117: (**A**) Hydrolysis of corncob powder by Y117—(**Left**) untreated substrate; (**Right**) substrate after enzymatic degradation, showing visible structural breakdown. (**B**,**C**) Colony morphology of Y117 grown on (**B**) PDA (potato dextrose agar) and (**C**) YPD (Yeast Extract–Peptone–Dextrose) media after 5 days at 30 °C. (**D**–**F**) Hyphal morphology during enzyme production—(**D**) pre-production phase, (**E**) mid-production phase, and (**F**) late-production phase. Scale bars: 20 μm (**D**,**E**); 10 μm (**F**).

Our laboratory isolated a wild-type strain from Jiangxi Province soil samples that demonstrated exceptional lignocellulose-degrading capability with high hydrolysis efficiency against various biomass substrates, including corn cob, rice straw, and ramie. *T. pinophilus* Y117, a mutant strain derived from the wild-type strain through random mutagenesis (Figure 1), was shown to produce a cellulase mixture with higher filter paperase (FPase), Avicelase, and β-glucosidase activity compared to previously reported strains, representing a valuable resource for large-scale production of bioenergy and biobased products [Table S1]. In this study, we performed whole-genome sequencing of the hyper-cellulolytic *T. pinophilus* strain Y117 and conducted systematic comparative genomic analysis with other *T. pinophilus* strains. This investigation was designed to (1) identify key genetic determinants associated with lignocellulose degradation, (2) elucidate strain-specific variations in degradation capabilities, and (3) uncover the molecular mechanisms underlying its superior cellulose-degrading efficiency. Our findings not only contribute to advancing the knowledge of the mechanistic basis of lignocellulose degradation by filamentous fungi but also establish a genetic framework for engineering *T. pinophilus* as a robust microbial cell factory for enhanced heterologous protein production.

## 2. Materials and Methods

### 2.1. Isolation and Culture Maintenance of T. pinophilus Y117

*T. pinophilus* Y117 is a hyper-cellulolytic mutant strain developed through successive mutagenesis of the wild-type TP117 isolated from paddy soil in Jiangxi Province, China. The strain development protocol involved sequential UV and chemical mutagenesis: The parental strain TP117 was first cultured on potato dextrose agar (PDA, Solarbio P8931) at 30 °C for 5 days, after which mycelial mats were harvested and homogenized in sterile distilled water to prepare a suspension adjusted to 10^8^ CFU/mL using a hemocytometer. For primary mutagenesis, 5 mL aliquots of this suspension were subjected to UV irradiation (254 nm wavelength) for 30 s at a distance of 10 cm, achieving 90% lethality as determined by viability plating. The irradiated cells were then plated on selective PDA medium containing 0.1% (*w*/*v*) AZCL-HE-cellulose (Megazyme, Wicklow, Ireland) and incubated at 30 °C for 72 h. Colonies demonstrating enhanced cellulolytic activity were identified by the formation of distinct blue halos (≥2 mm radius) resulting from chromogenic substrate hydrolysis. Potential mutants were validated through shake-flask cultivation in Mandels’ medium with 1% microcrystalline cellulose as a carbon source, followed by quantitative filter paper activity (FPA) assays performed according to IUPAC standard methods [22]. The top-performing UV mutant (TP117-UV9) showing the highest FPA was subsequently treated with 0.05% (*w*/*v*) N-methyl-N′-nitro-N-nitrosoguanidine (NTG) in phosphate buffer (50 mM, pH 7.0) for 30 min at room temperature, again calibrated to 90% lethality. Post-treatment, mycelia were recovered by centrifugation (8000× *g*, 4 °C, 10 min), washed twice with sterile phosphate buffer, and subjected to secondary screening on AZCL-HE-cellulose plates. The ultimate mutant Y117 was selected based on superior and stable cellulase production characteristics through three consecutive generations of cultivation. *T. pinophilus* Y117 was deposited at the China General Microbiological Culture Collection Center (CGMCC) under accession number CGMCC No. 42105. The strain was grown and maintained in potato dextrose agar (PDA) medium at 30 °C. A conidial suspension was stored in 25% glycerol at −80 °C and activated on PDA plates. The activated strain was inoculated on PDA medium at 30 °C for 4 days.

### 2.2. Cellulase Production and Enzyme Activity Assays

Cellulase production by *T. pinophilus* Y117 was evaluated using a modified protocol based on the method described by Ikeda et al. [23]. The strain was cultivated in a cellulase-inducing medium containing the following components (per liter): 50 g microcrystalline cellulose (CAS 9004-34-6; BBI Life Sciences, Shanghai, China) as the carbon source; 24 g KH_2_PO_4_; 1 g Tween 80; 5 g (NH_4_)_2_SO_4_; 1.2 g MgSO_4_·7H_2_O; 0.01 g ZnSO_4_·7H_2_O; 0.01 g MnSO_4_·6H_2_O; 0.01 g CuSO_4_·7H_2_O; 4 g urea. The medium pH was adjusted to 4.0 using 1 M H_2_SO_4_ or 1 M KOH prior to sterilization (121 °C, 20 min). Cultivation was performed in 250 mL Erlenmeyer flasks containing 100 mL of medium and incubated at 30 °C with agitation at 220 rpm for 7 days in an orbital shaker (ZHWY-211B; Shanghai Zhicheng Analytical Instrument Manufacturing Co., Ltd., Shanghai, China). Culture aliquots were withdrawn and centrifuged (10,000× *g*, 10 min, 4 °C) to remove mycelia and residual cellulose. The resulting supernatants were filtered (0.22 μm pore size) and stored at −20 °C as crude enzyme extracts for subsequent analyses.

Enzyme activities were determined in triplicate using standard spectrophotometric methods. Filter paper activity (FPase) was measured according to the method described by Ghose (1987) [22] using Whatman No. 1 filter paper as the substrate. One unit (U) of enzyme activity was defined as the amount of enzyme releasing 1 μmol of reducing sugars (glucose equivalent) per minute at 50 °C and pH 4.8. CMCase activity was assessed using carboxymethyl cellulose (CMC) as the substrate [24], expressed as 1 μmol reducing sugars produced per minute per mg soluble protein. Avicelase activity was determined using microcrystalline cellulose following the procedure described by Wood & Bhat (1988) [25]. Xylanase activity was measured using 1% (*w*/*v*) beechwood xylan in 50 mM sodium acetate buffer (pH 5.0) at 45 °C for 30 min according to established protocols [26]. β-glucosidase activity was quantified using 10 mM p-nitrophenyl-β-D-glucopyranoside (pNPG) in a 100 mM citrate buffer system at 45 °C for 10 min, with one unit corresponding to 1 μmol p-nitrophenol released per minute [27]. β-xylosidase activity was determined with 10 mM p-nitrophenyl-β-D-xylopyranoside (pNPX) under similar conditions, and absorbance at 420 nm was then measured. For all the assays, reducing sugars were quantified via the 3,5-dinitrosalicylic acid (DNS) method [28], and soluble protein concentration was determined by the Bradford assay [29] using bovine serum albumin (BSA) as the standard.

### 2.3. Hydrolysis of Corncob

The corncob powder (300 mesh, without additional pre-treatment) was enzymatically hydrolyzed in 50 mM acetate buffer (pH 4.8) with a solid loading of 20 wt% (1:5 *w*/*v* ratio). The crude cellulase extract obtained from *T. pinophilus* Y117 fermentation was added at a dosage of 1 mL per gram of dry substrate. The reaction mixture was incubated in a temperature-controlled orbital shaker at 50 °C and 100 rpm for 48 h. The released reducing sugars were measured using the 3,5-dinitrosalicylic acid (DNS) method [28]. Briefly, aliquots of hydrolysate were mixed with DNS reagent, heated in boiling water for 5 min, and cooled to room temperature. Absorbance was measured at 540 nm using a spectrophotometer, with glucose as the standard. The reducing sugar yield was expressed as grams of glucose equivalents per gram of dry corncob powder.

### 2.4. DNA Extraction, Sequencing, and Genome Assembly

Genomic DNA was extracted using Fungal Genomic DNA Extraction Kits (Guangzhou Magen Biotechnology Co., Ltd., Guangzhou, Guangdong, China) according to the manufacturer’s instructions. The DNA quality was assessed using Qubit (Thermo Fisher Scientific, Waltham, MA, USA) and Nanodrop (Thermo Fisher Scientific, Waltham, MA, USA) accordingly.

For sequencing on the PacBio platform (PacBio, Menlo Park, CA, USA), qualified genomic DNA was fragmented with G-tubes (Covaris, Woburn, MA, USA) and end-repaired to prepare SMRTbell DNA template libraries (with fragment size of >10 Kb selected by blue pippin system) according to the manufacturer’s specification. Library quality was assessed by Qubit^®^ 2.0 Flurometer (Life Technologies, Carlsbad, CA, USA), and average fragment size was estimated on a Bioanalyzer 2100 (Agilent, Santa Clara, CA, USA). Single-molecule real-time (SMRT) sequencing was performed on the Pacific Biosciences Hifi sequencer (PacBio, Menlo Park, CA, USA) according to the standard protocols.

Continuous long reads were generated from three SMRT sequencing runs. Reads longer than 500 bp with a quality value over 0.75 were merged together into a single dataset. Next, the hierarchical genome-assembly process (HGAP) pipeline [30] was used to correct for random errors in the long seed reads (seed length threshold 6 Kb) by aligning shorter reads from the same library against them. The resulting corrected, preassembled reads were used for de novo assembly using Celera Assembler (version 8.3) with an overlap-layout- consensus (OLC) strategy [31].

### 2.5. Genome Feature Prediction and Functional Annotation

Several genome features were predicted. Specifically, open reading frames (ORFs) were predicted using AUGUSTUS (v3.4.0) [32]. Repetitive elements were identified by RepeatMasker (v4.0.5) [33]. Prediction of noncoding RNAs, such as rRNAs, was carried out using rRNAmmer (v1.2) [34], while tRNAs were identified by tRNAscan (v1.3.1) [35].

Functional annotation of predicted genes was performed using an integrated multi-database approach. The sequences of predicted protein-coding genes were aligned against five major databases: (1) the NCBI non-redundant protein (Nr) database (downloaded May 2024), (2) UniProt/Swiss-Prot (release 2024_04), (3) Kyoto Encyclopedia of Genes and Genomes (KEGG, release 111), (4) Gene Ontology (GO), and (5) Clusters of Orthologous Groups for Eukaryotic Complete Genomes (KOG), assigning GO terms and pathway information.

### 2.6. Comparative Genomics and Phylogenetics Analysis

The genomic sequences of 11 *T. pinophilus* strains were retrieved from the NCBI database, while the genome of strain Y117 was newly sequenced in this study. Orthologous gene clusters among all 12 strains were identified using OrthoFinder (v2.5.4) [36] with default parameters. Single-copy orthologous genes were subsequently extracted for phylogenetic analysis. Multiple sequence alignment of these conserved genes was performed using MAFFT (v7.505) [37] to ensure accurate alignment of homologous sequences. To improve alignment quality, ambiguous regions and poorly aligned positions were removed using Gblocks (v0.91b) [38] with relaxed parameters.

A maximum-likelihood phylogenetic tree was reconstructed using FastTree (v2.1.11) [39] based on 5783 single-copy orthologous genes. Branch support values were estimated using the Shimodaira–Hasegawa test with 1000 resamples. The resulting phylogenetic tree was visualized and annotated using the version 7.1 of theInteractive Tree of Life (iTOL) web platform [40] to elucidate the evolutionary relationships among strain Y117 and the other *T. pinophilus* strains. The Gene Ontology (GO) enrichment analysis was performed using GOATOOLS (https://github.com/tanghaibao/GOatools, accessed on 18 February 2025) [41]. Fisher’s exact test was employed to determine statistically significant overrepresentation of GO terms among strain-specific genes compared to the background of all predicted genes in each strain. To control the false discovery rate (FDR) in multiple hypothesis testing, we applied four correction methods: Bonferroni, Holm, Sidak, and FDR (Benjamini–Hochberg). A GO term was considered significantly enriched if its corrected *p*-value was ≤0.05.

### 2.7. Identification of CAZymes, Secondary Metabolite Gene Clusters, and Transcription Factor

Carbohydrate-Active Enzymes (CAZymes) in *T. pinophilus* were predicted through comprehensive analysis against the CAZy database (www.cazy.org) [42] using BLASTp version 2.8.1 (E-value < 1 × 10^−5^) [43]. Proteins exhibiting >50% sequence identity and ≥90% query coverage were automatically assigned to CAZy families/subfamilies, whereas those with <50% identity were subjected to rigorous manual validation, including catalytic residue conservation analysis using InterProScan (v5.56-89.0) [44] and structural domain verification via HMMER (v3.3.2) against Pfam-A (v35.0) [45]. Concurrently, secondary metabolite biosynthetic gene clusters (BGCs) were predicted using antiSMASH (v6.1.1 fungal version) [46] with default parameters. Putative transcription factors (TFs) were identified by reciprocal BLASTp search against the Fungal Transcription Factor Database (FTFD; http://ftfd.snu.ac.kr/, accessed on 20 January 2025) [47], employing an E-value threshold of <1 × 10^−10^. Among the identified TFs, we selected 20 well-characterized TFs known to regulate biomass-degrading enzyme genes in filamentous fungi, based on established criteria [21].

### 2.8. Statistical Analysis and Visualization

Three replicates were used for each group. The SPSS software (v2.0) was used for data significance analysis, and the GraphPad Prism 9.5 software was used for plotting. Different lowercase letters indicate significant differences (*p* < 0.05).

### 2.9. Data Access

A total of 12 genome assemblies were analyzed in this study. One was newly sequenced (see Data Availability Statement). The remaining 11 genomes were retrieved from GenBank, under the accession numbers: *T. pinophilus* NCIM1228, GCA_033439165.1; *T. pinophilus* NA01, GCA_009805475.2; *T. pinophilus* AR155, GCA_011392495.1; *T. pinophilus* P8041, GCA_023623755.1; *T. pinophilus* M8004A, GCA_023623785.1; *T. pinophilus* J7Y3, GCA_023623735.1; *T. pinophilus* Y-94, GCA_000829775.1; *T. pinophilus* 1-95, GCA_001571465.2; *T. pinophilus* PSF-12(1), GCA_003316675.1; *T. pinophilus* NRRL3647, GCA_027569565.1; *T. pinophilus* NRRL3503, GCA_027569545.1.

## 3. Results

### 3.1. Assembly Statistics and General Features of T. pinophilus Y117 Genome

**Figure 2:**
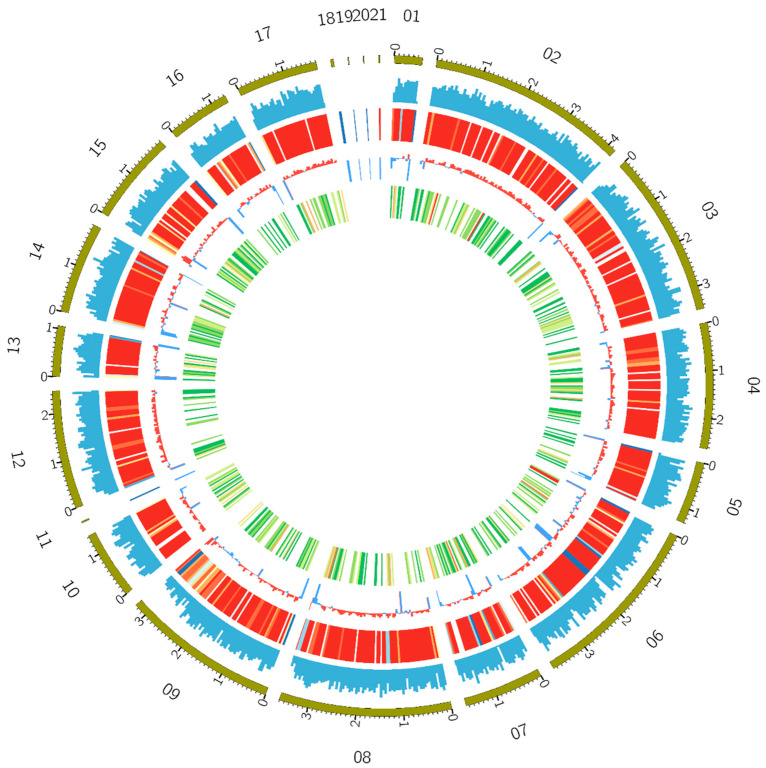
Overview of the Y117 genome assembly. The circle diagram from outside to inside represents different chromosome lengths, gene densities, repeat sequence densities (red to blue, representing the increase in sequence density), GC contents, and numbers of CAZyme genes in the window (white, green, yellow, and red, representing the increase in the number of genes). The first 21 scaffolds of the genome were taken for mapping using the Circos v0.69-3 software [48] with a window size of 50 kb.

The genome of *T. pinophilus* strain Y117 was sequenced using the PacBio Sequel II platform (PacBio, Menlo Park, CA, USA), generating a high-quality assembly with 98.9% completeness as assessed by BUSCO (version 5.7.1). The assembly comprised 21 contigs with an N50 contig length of 3.2 Mb and a maximum contig length of 4.2 Mb, exhibiting a GC content of 46.36% (Figure 2). Gene annotation predicted 12,406 protein-coding genes, along with 38 rRNA and 121 tRNA genes. The genome assembly was deposited in the NCBI genome database under BioProject ID: PRJNA1203055 (https://www.ncbi.nlm.nih.gov/bioproject/PRJNA1203055/, accessed on 15 August 2025). A summary of assembly and annotation statistics is shown in Table 1. Functional annotation using NCBI’s Clusters of Orthologous Groups (COG) database classified 5436 genes into eukaryotic orthologous groups (KOG categories) (Figure S1). The most gene-rich KOG categories included “General function prediction only”, “Secondary metabolites biosynthesis, transport and catabolism”, “Lipid transport and metabolism”, “Posttranslational modification, protein turnover, chaperones”, “Carbohydrate transport and metabolism”, “Energy production and conversion”, and “Signal transduction mechanisms” (Figure S1). The KEGG annotation results revealed that global and overview maps (555 genes), carbohydrate metabolism (155 genes), and amino acid metabolism (222 genes) were the most abundant categories in *T. pinophilus* Y117 (Figure S2).

### 3.2. Phylogenetic Tree and Comparative Genomics of T. pinophilus Strains

**Figure 3:**
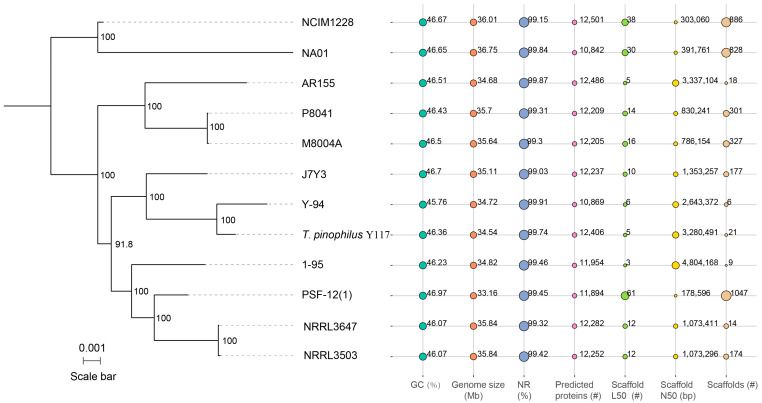
Phylogeny and genome statistics of Y117 and eleven other *T. pinophilus* strains. The phylogenetic tree was constructed using FastTree and MAFFT based on 5783 single-copy core genes (single-copy orthologs present in all strains). Seven bubble plots in the right panel illustrate key genomic features and sequencing quality parameters: GC content (%), genome size (Mbp), NR% (percentage of predicted proteins with BLASTP matches to NCBI Non-Redundant Protein Database), predicted protein count, scaffold L50 (number of scaffolds covering 50% of the genome), scaffold N50 (shortest scaffold length covering 50% of the genome), and total scaffolds. Bubble sizes are scaled within each panel and are not comparable across panels.

To elucidate the evolutionary relationships and genomic diversity within the *T. pinophilus* species, a comparative genomic analysis of twelve strains—including Y117 and eleven additional *T. pinophilus* strains accessed from the NCBI database (e.g., Y-94, 1-95, NRRL3503, NRRL3647, NCIM1228, etc.)—was conducted. These strains were isolated from diverse ecological niches—including soil, plant rhizospheres, animal feces, and synthetic fabric—providing a broad representation of the species’ genetic diversity. The assembled genomes exhibited an average size of 35.23 Mb and GC content of 46.41%, consistent with previously reported *Talaromyces* genomes (Figure 3). Notably, Y117 demonstrated superior assembly continuity, with only 21 scaffolds (Scaffold N50 = 3.28 Mb), far exceeding the contiguity of other strains such as PSF-12(1) (N50 = 178 kb) and NRRL lineages (average N50 = ~1.07 Mb). This high-quality assembly facilitates accurate gene annotation and downstream functional analyses. Furthermore, Y117 encodes 12,406 predicted proteins, a count comparable to those of high-yield strains NCIM1228 (12,501) and AR155 (12,486), and markedly higher than that of Y-94 (10,869). This expanded proteome may be associated with increased lignocellulolytic potential. Phylogenetic analysis of these 12 strains was performed based on 5783 single-copy core genes using FastTree and MAFFT. The resulting tree (scale = 0.001 substitutions per site) revealed that Y117 clusters most closely with Y-94, forming a distinct subclade separate from the other analyzed strains (Figure 3). The high bootstrap support (>90%) for all the major nodes confirms robust evolutionary relationships among these strains, with Y117’s unique phylogenetic position potentially associated with its exceptional lignocellulolytic phenotype.

### 3.3. Strain-Specific Genes

**Figure 4:**
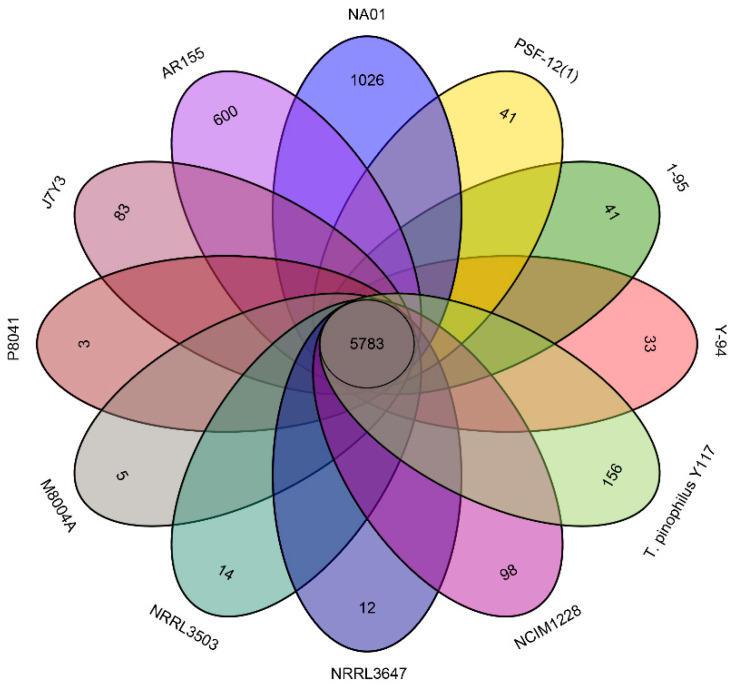
Venn diagram illustrating the numbers of homologous and strain-specific genes identified in the genomes of *T. pinophilus* strains. Genes were classified as strain-specific if they had <50% coverage and/or <50% similarity to the nucleotide sequences of all genes in the other strains.

Strain-specific genes were identified among the predicted genes in the genomes of the twelve *T. pinophilus* strains. These genes were defined as meeting either of the following criteria: (1) <50% sequence similarity, or (2) <50% coverage when aligned with nucleotide sequences of genes from the other sequenced strains (whichever criterion was more stringent). Based on these criteria, more than 5783 genes were identified as homologous (Figure 4). The genome of *T. pinophilus* NA01 had the highest number of strain-specific genes (1026, Figure 4), followed by *T. pinophilus* AR155 (600) and *T. pinophilus* Y117 (156). An enrichment analysis was conducted to compare the strain-specific genes of an isolate with all the predicted genes of the same isolate. The analysis revealed that *T. pinophilus* NA01 exhibits significant enrichment in the GO terms related to fungal polarized growth and cellular morphogenesis, including “cellular bud” (GO:0005933), “site of polarized growth” (GO:0030427), and “cellular bud neck” (GO:0005935) (Figure S3). The GO enrichment analysis of strain-specific genes in strain AR155 revealed significant enrichment in biological processes related to cellular morphogenesis and genome maintenance (Figure S4). The most significantly enriched terms included ‘regulation of cell shape’ (GO:0008360), ‘regulation of cell morphogenesis’ (GO:0008632), ‘regulation of developmental process’ (GO:0050793), and ‘DNA metabolic process’ (GO:0006259). The genome of *T. pinophilus* Y117 is only represented in the GO term “organonitrogen compound biosynthetic process” (GO:1901566), which suggests distinct adaptations in nitrogen metabolism compared to other related strains.

### 3.4. The CAZymes Among T. pinophilus Strains

**Figure 5:**
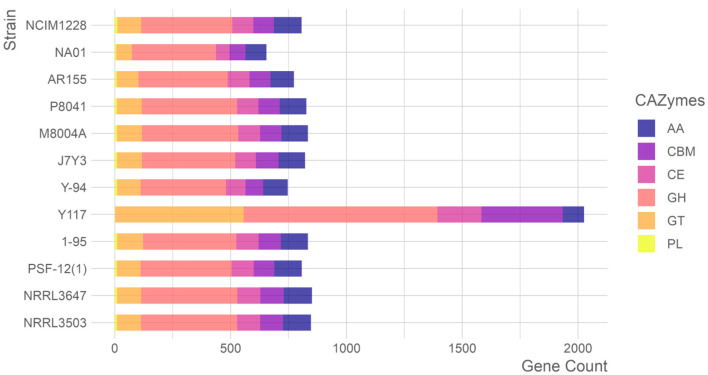
Carbohydrate-active enzymes in the twelve *T. pinophilus* strains, sorted according to the phylogram of Figure 1. Abbreviation: carbohydrate-binding module (CBM), carbohydrate esterase (CE), glucoside hydrolase (GH), glycosyl transferase (GT), polysaccharide lyase (PL), and auxiliary activity (AA).

Comprehensive analysis of carbohydrate-active enzymes (CAZymes) across *T. pinophilus* strains revealed 2026 CAZyme-encoding genes within the 12,406 predicted proteins of *T. pinophilus* Y117. These genes spanned six functional classes, including 75 glycoside hydrolase (GH) families, 49 glycosyltransferase (GT) families, 14 carbohydrate esterase (CE) families, 34 carbohydrate-binding module (CBM) families, 9 auxiliary activity (AA) families, and 2 polysaccharide lyase (PL) families. Comparative genomic analysis with eleven additional *T. pinophilus* strains identified significant strain-specific variations in CAZyme gene content, highlighting the enzymatic diversity within this species (Figure 5). Notably, Y117 exhibited a substantial expansion of GH genes (*n* = 837), exceeding the other strains (363–416 genes) by 101–130%. Similarly, its CBM gene repertoire (*n* = 351) was 3.5–4.6 times larger than that of comparator strains (77–101 genes). These genomic features suggest elevated capacity for both glycosidic bond hydrolysis and carbohydrate substrate recognition in Y117. Conversely, Y117 showed reduced gene complements in AA (9 families) and PL (2 families) categories compared to the other strains, potentially reflecting specialized adaptations in oxidative and lytic enzymatic pathways. The exceptional enrichment of GH and CBM genes in Y117 correlates with its demonstrated efficiency in plant biomass degradation, while the relative paucity of AA and PL genes may indicate niche-specific metabolic specialization.

**Figure 6:**
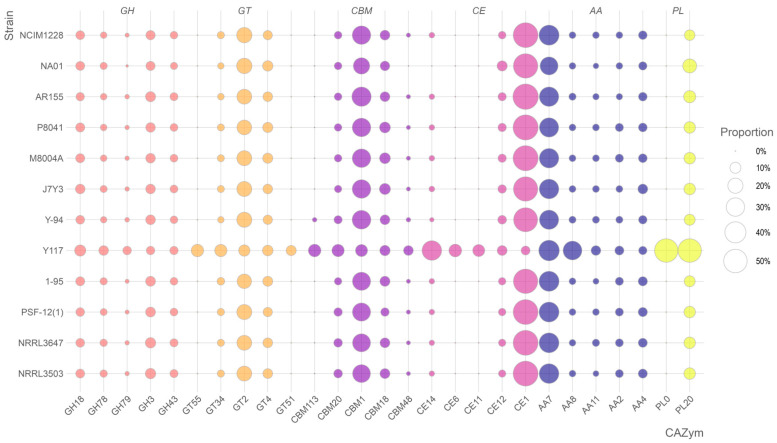
Bubble plot depicting the CAZyme profiles of the twelve *T. pinophilus* strains. For each CAZyme category, the five most prevalent clusters identified in strain Y117 were compared across all strains, if applicable. The size of each bubble indicates the proportion of genes in the cluster among all the genes belonging to the specific CAZyme category, and the colors indicate different CAZyme categories.

The comparative genomic analysis of carbohydrate-active enzyme (CAZyme) distributions across twelve fungal strains revealed striking enzymatic specialization in strain Y117, which exhibits an exceptionally expanded repertoire of glycoside hydrolases (GHs), carbohydrate esterases (CEs), and glycosyltransferases (GTs), distinguishing it as a metabolic outlier with unique biotechnological potential (Figure 6). Y117 demonstrated remarkable expansion in three key GH families—GH18 (10.39% of total GHs, 1.7-fold higher than the 6.24 ± 0.72% average of the other strains), GH78 (7.53%, 2.2-fold higher than the 3.48 ± 0.41% average), and GH79 (5.73%, 5.8-fold higher than the 0.98 ± 0.38% average)—while control strains like Y-94 and NCIM1228 displayed typical ascomycete GH distributions dominated by GH3 (cellulases; 6.8 ± 0.9%) and GH43 (xylanases; 4.3 ± 0.5%). In the CE family, Y117 showed unprecedented dominance of CE14 (34.21% of total CEs, 30.5-fold higher than the 1.12 ± 0.38% average) and CE6 (13.68%, 6.1-fold higher than the 2.25 ± 0.42% average), suggesting enhanced capacity for deacetylating xylan and chitin, whereas other strains exhibited conserved CE1 dominance (54.2 ± 3.8%). Y117’s GT profile further distinguished it through complete GT55/GT34/GT51 suites (32.3% of GTs versus <8% in other strains), including GT55 (12.99%, absent in other strains), indicating potential innovation in α-glucan synthesis. The co-occurrence of hyper-expressed CE14 with enhanced GH78/GH79 pectinases (12.8% of GHs) suggests a coordinated strategy for degrading complex plant cell walls, positioning Y117 as a strain with potential for biomass conversion, biofuel production, and processing of recalcitrant polysaccharides.

### 3.5. Secondary Metabolite (SM) Biosynthetic Potential Across T. pinophilus Strains

**Figure 7:**
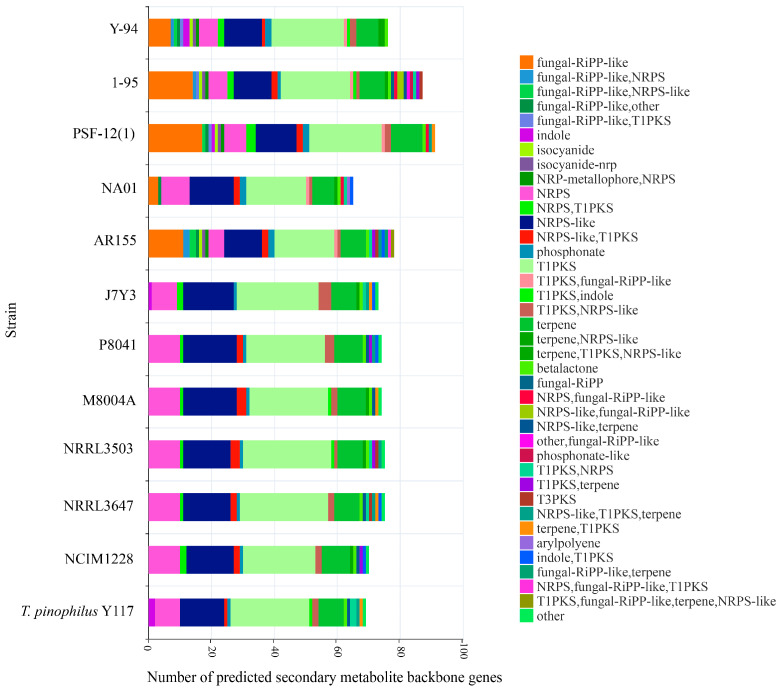
Core biosynthetic genes in *Talaromyces pinophilus* strains predicted by antiSMASH. Abbreviations: RiPP (ribosomally synthesized and post-translationally modified peptide), NRPS (non-ribosomal peptide synthase), T1PKS (type I polyketide synthase), and T3PKS (type III polyketide synthase).

Comprehensive analysis of SM biosynthetic gene clusters (BGCs) was performed using fungal antiSMASH v6.0.1, revealing distinct strain-specific metabolic profiles (Table S2). *T. pinophilus* Y117 exhibited a streamlined SM repertoire, encoding only 69 backbone genes—the second lowest among all the strains after NA01 (Figure 7). This represents a 38.9% reduction compared to the most gene-rich strain, NRRL3503 (113 backbone genes), suggesting evolutionary selection for metabolic economy in Y117. The conserved core SM pathways present in all the strains included Type I polyketide synthases (T1PKSs) (19–28 clusters per strain), non-ribosomal peptide synthetases (NRPSs) (6–10 clusters), NRPS-like enzymes (12–17 clusters), and ubiquitous betalactone clusters (1 per genome). These evolutionarily maintained pathways likely support essential functions. Fungal-RiPP-like clusters displayed higher diversity in strain Y-94 and 1-95 (Y-94:7; 1-95:14), while these were completely absent in Y117, J7Y3, P8041, M8004A, and NRRL strains. While most strains possessed NRPS-T1PKS hybrids (0–3 clusters), Y117 uniquely lacked these but contained two indole and two T1PKS-NRPS clusters, reflecting divergent metabolic specialization. The complete absence of fungal-RiPP-like clusters in Y117, coupled with its simplified NRPS/T1PKS profile, could indicate adaptive genome streamlining favoring hydrolytic enzyme production over secondary metabolic complexity.

### 3.6. Analysis of Transcription Factors in T. pinophilus

**Figure 8:**
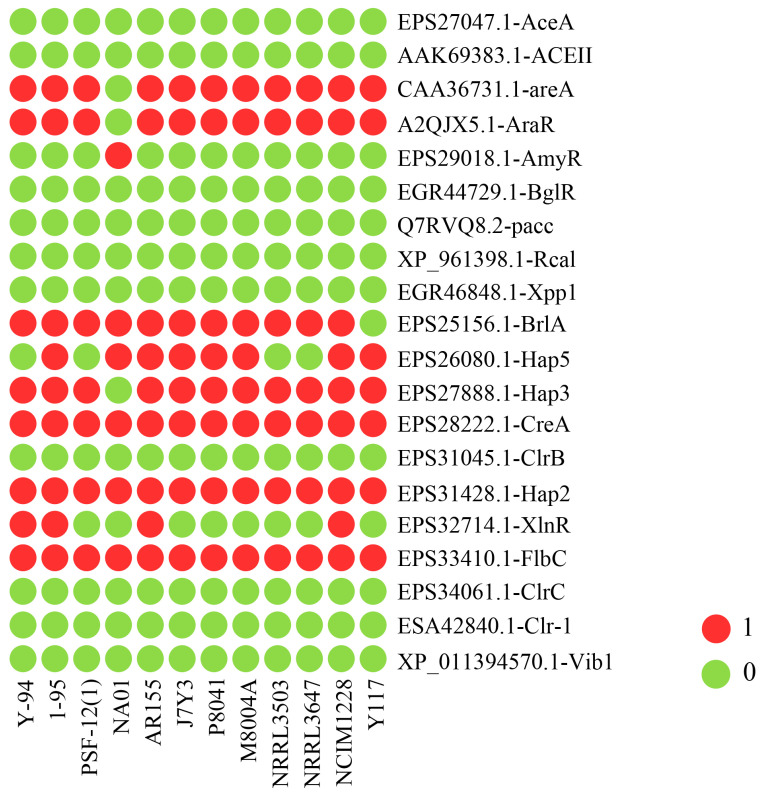
Comparative genomic analysis of transcription factors regulating biomass-degrading enzyme genes across twelve *Talaromyces pinophilus* strains.

Transcription factors (TFs) play a crucial role in regulating gene expression and cellular processes, significantly influencing metabolite levels. To explore their potential regulatory contributions to strain-specific diversity in cellulose degradation, we compared the presence or absence of specific transcription factor (TF) genes among the twelve *T. pinophilus* strains (Figure 8). Several TF genes, such as CAA36731.1-areA, A2QJX5.1-AraR, EPS27888.1-Hap3, EPS28222.1-CreA, and EPS33410.1-FlbC, were identified in all strains, including Y117, indicating their conserved roles in fungal biology. Although Y117 shares most TF genes with other strains, with no TF genes uniquely present in this strain, several differences between Y117 and other strains have been observed. For example, EPS32714.1-XlnR is absent in Y117 but present in some other strains (e.g., Y-94, 1-95, AR155, and NCIM1228), indicating a possible difference in the regulation of xylanase-related pathways. The TF gene EPS25156.1-BrlA, which plays a key role in the initiation of asexual development in fungi, is present in all the strains except Y117, suggesting a potential loss or divergence in this regulatory pathway in Y117.

## 4. Discussion

*Talaromyces pinophilus* is a filamentous fungus renowned for its exceptional lignocellulose-degrading capabilities, attributed to its robust enzymatic machinery and potential as a microbial cell factory for industrial applications. The mutant strain *T. pinophilus* Y117, derived from the wild-type parental strain TP117 through UV and chemical mutagenesis, demonstrates superior enzymatic productivity, secreting a comprehensive cellulolytic enzyme system including filter paperase (FPase), carboxymethyl cellulase (CMCase), avicelase, β-glucosidase, xylanase, and xylosidase (Table S1). The outstanding lignocellulose-degrading capacity of Y117 can be traced to its parental strain, TP117, which was originally isolated from paddy soil in Jiangxi Province, China. In this region, rice is cultivated twice a year (double-cropping). As part of local farming practices, after each harvest, rice straw is crushed and returned to the fields as an organic carbon source. This practice has created an environment conducive to the enrichment of lignocellulose-degrading fungi in the paddy soil. Notably, combined UV and chemical mutagenesis has been demonstrated as a highly effective strategy for enhancing cellulase production in fungi [49,50]. The enzymatic profile of Y117 is particularly robust due to its balanced secretion of multiple cellulolytic and hemicellulolytic enzymes, making it an outstanding candidate for biomass conversion applications.

In this study, we performed whole-genome sequencing and comprehensive functional annotation of this strain. Additionally, we conducted a comparative genomic analysis using all publicly available whole-genome sequences of *T. pinophilus* strains. Our findings revealed that Y117 possesses a genomically streamlined architecture, characterized by high assembly continuity and strong coding potential, features that make it an ideal candidate for mechanistic studies on lignocellulose degradation (fewer scaffolds enable more accurate gene mapping). Gene annotation identified 12,406 protein-coding genes in Y117, a number significantly greater than that of the well-characterized cellulase-producing strain Y-94, suggesting elevated enzymatic potential for lignocellulose deconstruction. Although phylogenetic analysis confirmed a close relationship between Y117 and Y-94, Y117 exhibited distinct genomic features, which likely reflect specialized metabolic and regulatory adaptations crucial for its ecological niche. These unique genomic traits may underpin its superior lignocellulolytic efficiency and could help unravel strain-specific optimization strategies.

Genomic analysis of strain Y117 identified 2026 CAZymes, representing a remarkably rich repertoire compared to the average of 800 observed in other reference *T. pinophilus* strains (Figure 5). This extensive CAZyme complement includes numerous enzymes with demonstrated biomass-hydrolyzing potential. The comparative analysis of CAZyme distribution between Y117 and Y-94 revealed striking functional specialization in Y117, particularly within the glycoside hydrolase (GH) families, where GH78 (7.5%) and GH79 (5.7%) were significantly more abundant than in Y-94. While GH78 (α-L-rhamnosidase) primarily targets pectin, its activity could indirectly facilitate cellulose access by removing rhamnogalacturonan barriers in plant cell walls [51]. GH79 enzymes cleave β-glucuronidic linkages in glycosaminoglycans and plant polysaccharides [52]. The combination of GH78 (pectin-targeting) and GH79 (glucuronide-targeting) may synergistically improve Y117’s cellulose degradation efficiency, as demonstrated by its higher FPase (1.32 U/mg) and β-glucosidase (3.22 U/mg) activities [Table S1] compared to Y-94, through the dismantling of complex plant cell walls or extracellular matrices. This multifunctional approach designates Y117 as a robust lignocellulose degrader, where GH78/GH79 may play indirect but critical roles in enhancing cellulose accessibility and hydrolysis. Strain Y117 demonstrates a unique and highly expanded CE profile (Figure 6). Most remarkably, Y117 showed an exceptional representation of CE14 and CE6, corresponding to 30.5-fold and 6.1-fold increases, respectively, over the average of the other strains. CE14 enzymes primarily remove acetyl groups from plant polysaccharides (e.g., xylan or chitin), enhancing their accessibility to glycoside hydrolases (GHs) for degradation [53]. The co-occurrence of hyper-expressed CE14 with the strain’s enhanced GH78/GH79 pectinases indicates a coordinated strategy for tackling complex plant cell walls, positioning Y117 as a promising candidate for biotechnological applications in biomass conversion and biofuel production. Y117’s GT55 expansion likely reflects niche adaptation for polysaccharide modification, as these glycosyltransferases are rare in related strains. Their predicted role in α-glucan synthesis may contribute to both self-tolerance during lignocellulose breakdown and production of modified oligosaccharides with industrial applications [54,55]. The strain’s GT55 profile warrants functional characterization to validate these hypothesized mechanisms [56].

Comparative genomic analysis of *T. pinophilus* strains revealed a specialized secondary metabolite profile of Y117 marked by the complete absence of fungal-RiPP-like clusters—a striking contrast to related strains such as Y-94 (7 clusters) and 1-95 (14 clusters). Genomic reduction in Y117, consistent with the streamlining phenomenon observed in industrial fungi, parallels its exceptional enrichment in cellulolytic enzymes (e.g., GH78 endoglucanases and CBM13 cellulose-binding modules) [57,58,59]. We propose this represents an adaptive metabolic trade-off where resource allocation favors lignocellulose degradation over specialized metabolite biosynthesis, a pattern documented in other high-performance cellulolytic fungi [54,60]. Notably, Y117 retains key biosynthetic capabilities, including 8 NRPS clusters, 25 T1PKS clusters, and unique T1PKS-NRPS hybrids (*n* = 2, absent in most strains). The lack of fungal-RiPPs may reflect Y117’s adaptation to nutrient-rich environments where competitive antimicrobials are less critical, while its retained NRPS/PKS systems could fortify stress tolerance and/or support niche colonization. These features collectively identify Y117 as an exceptional chassis for cellulase production, where redirected metabolic flux from secondary metabolism to hydrolytic enzyme synthesis could explain its superior biomass degradation performance (Figure 1). Future studies should validate this model through targeted approaches, including (1) time-course transcriptomic analysis under industrial fermentation conditions, (2) comparative promoter activity assays of key hydrolase genes, and (3) metabolic flux analysis during different growth phases.

TFs are proteins that control the transcription of target genes through binding to specific DNA sequence elements such as promoters, enhancers, etc. Elucidating the regulatory mechanisms of TFs coupled with synthetic biology-driven engineering of high-performance expression systems could significantly increase the industrial utilization of cellulolytic enzymes. Similar strategies have been successfully applied to improve enzyme production [61]. The transcription factor BrlA serves as a crucial developmental regulator in fungi, particularly within the *Aspergillus* genus, where it governs the formation of asexual spores (conidia) [62]. Loss of BrlA function is known to completely abolish conidiation. In the context of our study, the absence of the BrlA gene in *T. pinophilus* Y117 likely triggers metabolic reallocation, whereby resources typically dedicated to sporulation are redirected toward other biosynthetic pathways. This shift may account for the observed enhancement in cellulase production (Table S1). Such metabolic reprogramming aligns with established fungal adaptation strategies, wherein disruptions in developmental pathways often lead to compensatory upregulation of secondary metabolic or secretory processes [63]. Notably, this phenomenon has been documented in *Aspergillus nidulans*, where BrlA-deficient strains exhibit altered carbon flux toward secondary metabolite synthesis [64]. The Y117 phenotype thus exemplifies how targeted genetic modifications can exploit endogenous regulatory networks to optimize industrial enzyme yields. The transcription factor XlnR is a key transcriptional regulator of xylanolytic and cellulolytic enzyme genes in fungi [65]. Knockout of XlnR typically leads to a significant reduction in xylanase production, while its impact on cellulase activity tends to be less pronounced [66]. Consistent with this paradigm, our findings demonstrate that the absence of the XlnR gene in the Y117 genome aligns with the strain’s observed enzymatic profile [Table S1]. This phenotypic consistency reinforces the hierarchical regulatory function of XlnR, where its loss disproportionately affects xylanases over cellulases. Overall, these findings offer novel insights into the regulatory mechanisms underlying fungal plant-biomass-degrading enzymes and identify potential targets for genetic engineering to enhance industrial applications.

## 5. Conclusions

In conclusion, this study presents a high-quality genome assembly of *T. pinophilus* Y117, revealing genomic adaptations that underlie its exceptional cellulolytic performance. Comparative genomics highlighted three key evolutionary signatures: (1) significant expansion of GH and CBM families enabling efficient lignocellulose hydrolysis, (2) specialized secondary metabolism evidenced by fungal-RiPP cluster loss but retention of unique T1PKS-NRPS/indole clusters, and (3) BrlA gene deletion likely redirecting metabolic flux from sporulation to cellulase production. These findings not only provide a deeper understanding of fungal CAZyme regulation but also identify Y117 as an ideal chassis for industrial enzyme production, with its genomic features offering specific targets for strain engineering aimed at optimizing biomass degradation.

## Figures and Tables

**Table 1:** Genome features of *T. pinophilus* Y117.

Genome Features	Value
Size of assembled genome (Mbp)	36.52
GC content of assembled genome (%)	46.36
N50 length (bp)	3,280,491
N90 length (bp)	1,325,600
Maximum length (bp)	4,185,411
Minimum length (bp)	14,568
All protein-coding genes	12,406
Protein-coding genes (≥60 aa)	12,392
Average gene length (bp)	1588.09
Average number of introns per gene	1.89
Average intron length (bp)	132.83
Average number of exons per gene	2.89
Average exon length (bp)	1455.26
tRNA genes	121
rRNA genes	38

## Data Availability

All data are available in Section 3 and Supplementary Materials. The genomic data of Y117 are available in the NCBI database at https://api.ncbi.nlm.nih.gov/datasets/v2/genome/accession/GCA_048181935.1/download?include_annotation_type=GENOME_FASTA&include_annotation_type=GENOME_GFF&include_annotation_type=RNA_FASTA&include_annotation_type=CDS_FASTA&include_annotation_type=PROT_FASTA&include_annotation_type=SEQUENCE_REPORT&hydrated=FULLY_HYDRATED (accessed on 27 February 2025) under accession number SAMN45969786.

## Supplementary Materials

The following supporting information can be downloaded at https://www.mdpi.com/article/10.3390/jof11090614/s1, Figure S1: KOG categories in the *T. pinophilus* strain Y117 genome. Figure S2: Distribution of genes based on KEGG categories in the genomes of *T. pinophilus* strain Y117. Figure S3: GO enrichment analysis of strain-specific genes in *T*. *pinophilus* NA01. Figure S4: GO enrichment analysis of strain-specific genes in *T*. *pinophilus* AR155. Table S1: Specific enzyme activities of crude enzyme produced by *T. pinophilus* strain Y-94, strain CF-2612, and strain Y117. Table S2: Distribution of transcription factor genes among twelve *T. pinophilus* strains.
