# Supplementary material for: Genome Sequencing and Comparative Genomics of the Hyper-Cellulolytic Fungus Talaromyces pinophilus Y117
Source: J Fungi (Basel). 2025 Aug 22;11(9):614. doi: 10.3390/jof11090614 (PMC12471171; doi:10.3390/jof11090614)
Supplement: Supplementary file 1 [file jof-11-00614-s001.zip › jof-3798142-supplementary.pdf]

Supplementary Material

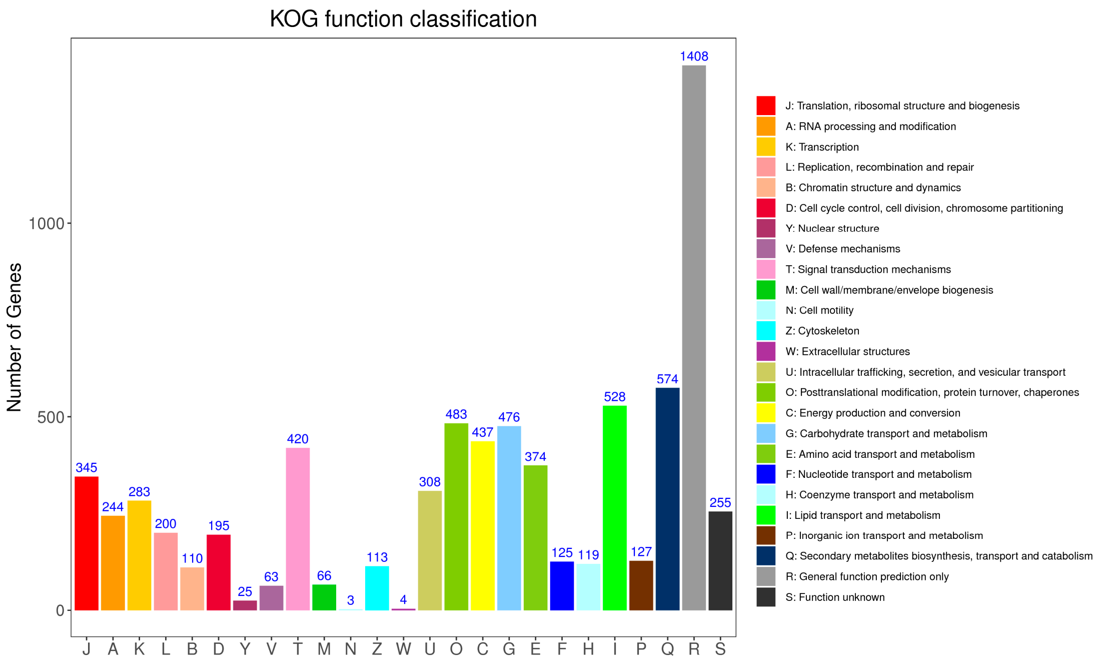

Figure S1. KOG categories in the *T. pinophilus* strain Y117 genome. The number on the top of the categories represents the number of genes.

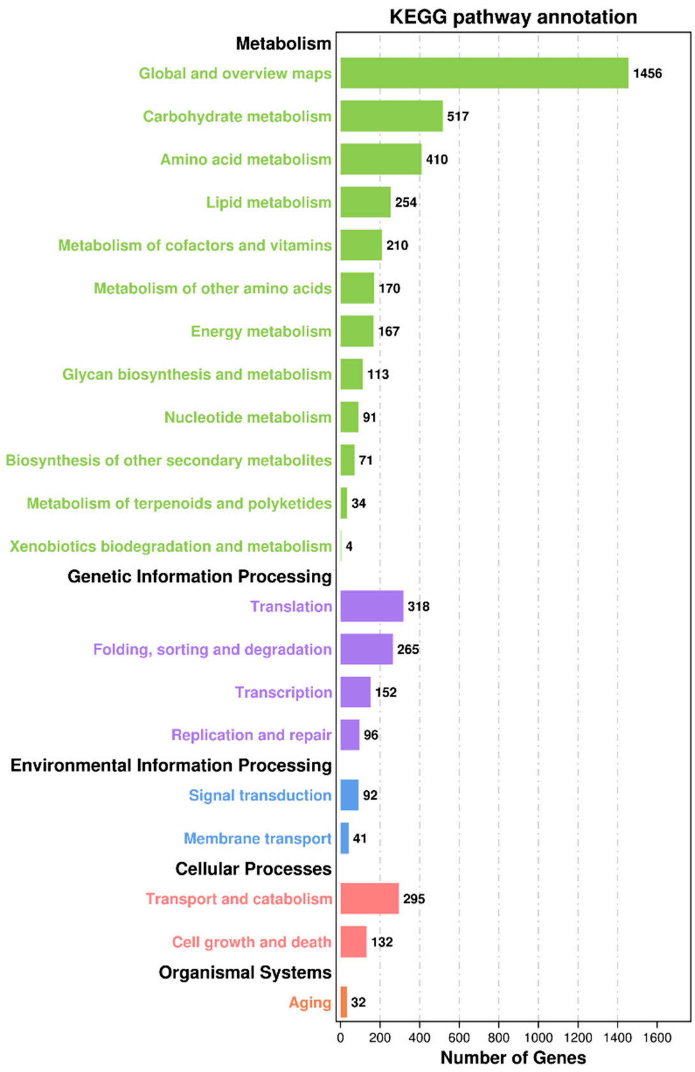

Figure S2. Distribution of genes based on KEGG categories in the genomes of *T. pinophilus* strain Y117. The number on the right of the categories represents the number of genes.

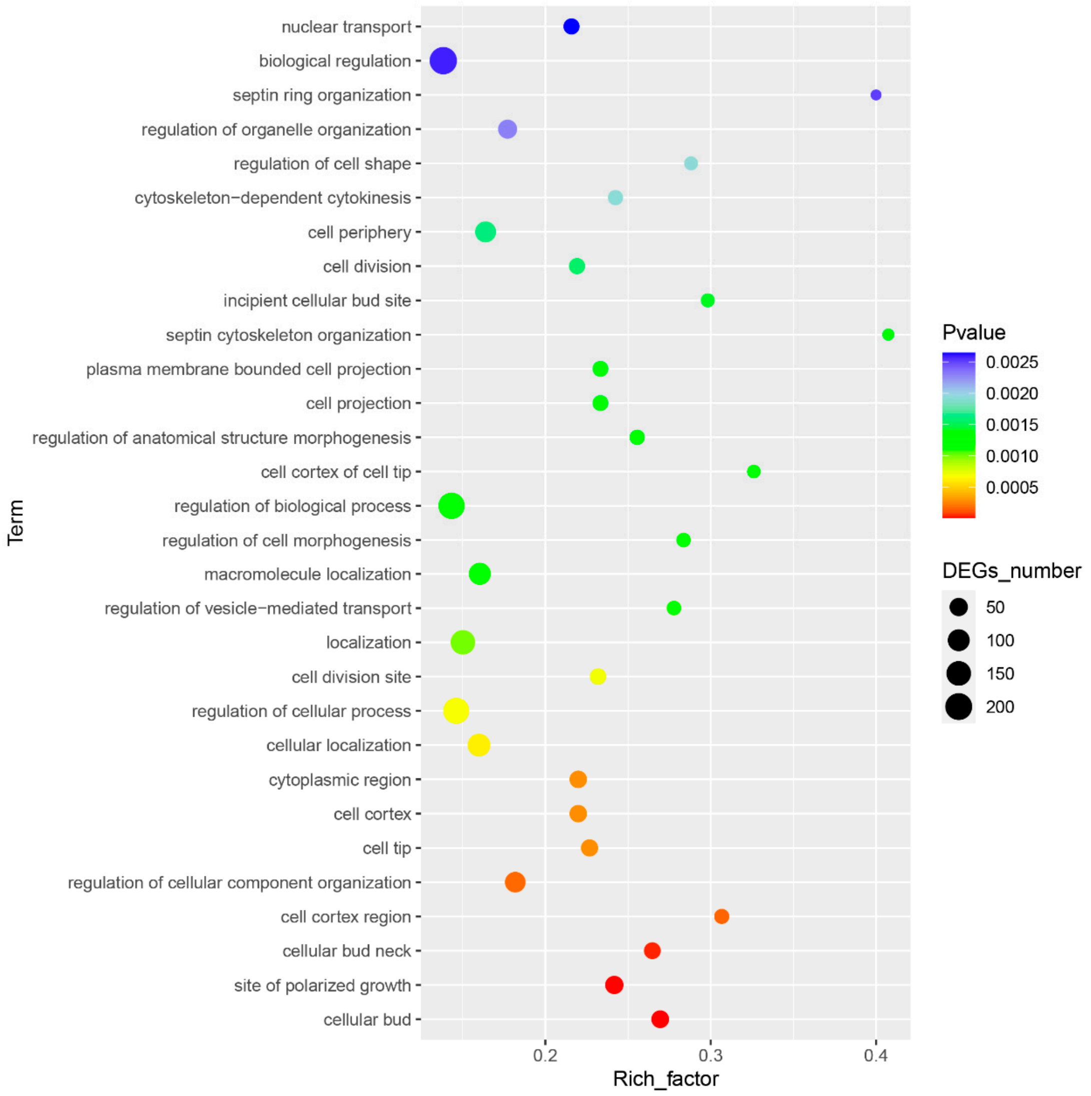

Figure S3. GO enrichment analysis of strain-specific genes in *T. pinophilus* NA01. The Rich factor (x-axis) represents the enrichment level of each term, while the y-axis lists the GO terms. The dot color gradient (blue to red) indicates the adjusted p-value (ranging from 0.0025 [blue] to 0.0005 [red]). Dot size corresponds to the number of DEGs (50–200) linked to each term.

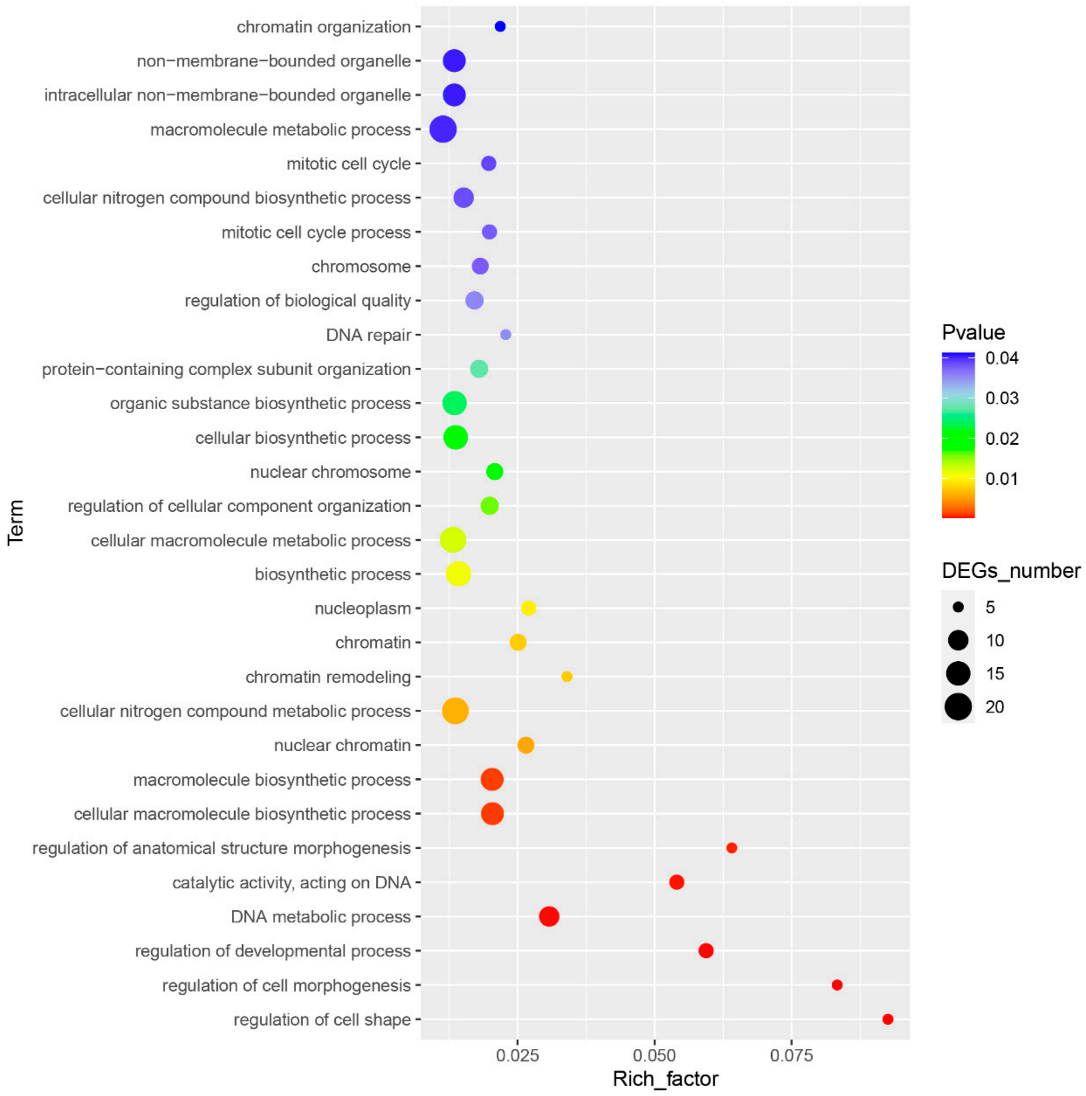

Figure S4. GO enrichment analysis of strain-specific genes in *T. pinophilus* AR155. The Rich factor (x-axis) represents the enrichment level of each term, while the y-axis lists the GO terms. The dot color gradient (blue to red) indicates the adjusted p-value (ranging from 0.04 [blue] to 0.01 [red]). Dot size corresponds to the number of DEGs (5–20) linked to each term.

Table S1. Specific enzyme activities of crude enzyme produced by *T. pinophilus* strain Y-94, strain CF-2612 and strain Y117 in the cellulose medium over 7days at 30 °C in shake culture.

Analysis items		Y-94	CF-2612	Y117
Specific enzyme activity (U/mg soluble protein)	FPase	0.51	0.98	1.32
	CMCase	8.72	4.56	5.67
	Avicelas	0.1	0.35	0.68
	β-glucosidas	1.23	2.26	3.22
	Xylanase	64	29.7	31.5
	Xylosidase	0.1	0.02	0.08

Table S2. Distribution of transcription factor genes among twelve *T. pinophilus* strains.

Transcription factor	Y-94	1-95	PSF-12(1)	NA01	AR155	J7Y3	P8041	M8004A	NRRL 3503	NRRL 3647	NCIM 1228	Y117
fungal-RiPP-like	7	14	17	3	11	0	0	0	0	0	0	0
fungal-RiPP-like, NRPS	1	1	0	0	2	0	0	0	0	0	0	0
fungal-RiPP-like, NRPS-like	1	0	1	0	2	0	0	0	0	0	0	0
fungal-RiPP-like,other	1	0	1	1	1	0	0	0	0	0	0	0
fungal-RiPP-like, T1PKS	1	1	1	0	0	0	0	0	0	0	0	0
indole	2	0	1	0	0	1	0	0	0	0	0	2
isocyanide	1	1	1	0	1	0	0	0	0	0	0	0
isocyanide-nrp	1	1	1	0	1	0	0	0	0	0	0	0
NRP-metallophore,NRPS	1	1	1	0	1	0	0	0	0	0	0	0
NRPS	6	6	7	9	5	8	10	10	10	10	10	8
NRPS, T1PKS	2	2	3	0	0	2	1	1	1	1	2	0
NRPS-like	12	12	13	14	12	16	17	17	15	15	15	14
NRPS-like, T1PKS	1	2	2	2	2	0	2	3	3	2	2	1
phosphonate	2	1	2	2	2	1	1	1	1	1	1	1
T1PKS	23	22	23	19	19	26	25	25	28	28	23	25
T1PKS, fungal-RiPP-like	1	1	1	1	1	0	0	0	0	0	0	0
T1PKS, indole	1	1	0	0	0	0	0	1	1	0	0	1
T1PKS, NRPS-like	2	1	2	1	1	4	3	2	1	2	2	2
terpene	7	8	10	7	8	8	9	9	8	8	9	8
terpene, NRPS-like	1	0	0	0	0	1	0	0	1	0	0	0
terpene, T1PKS, NRPS-like	1	1	0	1	0	0	0	1	0	0	1	0
betalactone	1	1	1	1	1	1	1	1	1	1	1	1
fungal-RiPP	0	1	0	0	0	0	0	0	0	0	0	0
NRPS, fungal-RiPP-like	0	1	1	1	0	0	0	0	0	0	0	0
NRPS-like, fungal-RiPP-like	0	2	0	0	0	0	0	0	0	0	0	0
NRPS-like, terpene	0	1	0	0	0	0	1	1	0	1	1	1
other, fungal-RiPP-like	0	1	0	0	0	0	0	0	0	0	0	0
phosphonate-like	0	1	0	0	0	0	0	0	0	0	0	0

T1PKS, NRPS	0	1	0	1	1	1	0	0	1	1	0	2
T1PKS, terpene	0	1	0	0	1	0	1	0	1	0	1	0
T3PKS	0	1	0	0	1	0	0	0	1	1	0	0
NRPS-like, T1PKS, terpene	0	0	1	0	1	1	1	0	1	1	0	1
terpene, T1PKS	0	0	1	0	0	1	0	1	0	1	0	1
arylpolyene	0	0	0	1	0	0	0	0	0	0	0	0
indole, T1PKS	0	0	0	1	1	1	1	0	0	1	1	0
fungal-RiPP-like, terpene	0	0	0	0	1	0	0	0	0	0	0	0
NRPS, fungal-RiPP-like, T1PKS	0	0	0	0	1	0	0	0	0	0	0	0
T1PKS, fungal-RiPP-like, terpene, NRPS-like	0	0	0	0	1	0	0	0	0	0	0	0
other	0	0	0	0	0	1	1	1	1	1	1	1
